# Global comparison of chromosome X genes of pulmonary telocytes with mesenchymal stem cells, fibroblasts, alveolar type II cells, airway epithelial cells, and lymphocytes

**DOI:** 10.1186/s12967-015-0669-8

**Published:** 2015-09-28

**Authors:** Yichun Zhu, Minghuan Zheng, Dongli Song, Ling Ye, Xiangdong Wang

**Affiliations:** Zhongshan Hospital, Shanghai Institute of Clinical Bioinformatics, Fudan University Center for Bioinformatics, Fudan University, Shanghai, China

**Keywords:** Chromosome X, Genes, Lung, Telocytes, Mesenchymal stem cells, Fibroblasts, Alveolar type II cells, Airway epithelial cells, Lymphocytes

## Abstract

**Background:**

Telocytes (TCs) are suggested as a new type of interstitial cells with specific telopodes. Our previous study evidenced that TCs differed from fibroblasts and stem cells at the aspect of gene expression profiles. The present study aims to search the characters and patterns of chromosome X genes of TC-specific or TC-dominated gene profiles and fingerprints, investigate the network of principle genes, and explore potential functional association.

**Methods:**

We compared gene expression profiles in chromosome X of pulmonary TCs with mesenchymal stem cells (MSC), fibroblasts (Fb), alveolar type II cells (ATII), airway basal cells (ABC), proximal airway cells (PAC), CD8^+^ T cells come from bronchial lymph nodes (T-BL), or CD8^+^ T cells from lungs (T-L) by global analyses, and selected the genes which were consistently up or down regulated (>1 fold) in TCs compared to other cells as TC-specific genes. The functional and characteristic networks were identified and compared by bioinformatics tools.

**Results:**

We selected 31 chromosome X genes as the TC-specific or dominated genes, among which 8 up-regulated (Flna, Msn, Cfp, Col4a5, Mum1l1, Rnf128, Syn1, and Srpx2) and 23 down-regulated (Abcb7, Atf1, Ddx26b, Drp2, Fam122b, Gyk, Irak1, Lamp2, Mecp2, Ndufb11, Ogt, Pdha1, Pola1, Rab9, Rbmx2, Rhox9, Thoc2, Vbp1, Dkc1, Nkrf, Piga, Tmlhe and Tsr2), as compared with other cells.

**Conclusions:**

Our data suggested that gene expressions of chromosome X in TCs are different with those in other cells in the lung tissue. According to the selected TC-specific genes, we infer that pulmonary TCs function as modulators which may enhance cellular growth and migration, resist senescence, protect cells from external stress, regulate immune responses, participate in tissue remodeling and repair, regulate neural function, and promote vessel formation.

**Electronic supplementary material:**

The online version of this article (doi:10.1186/s12967-015-0669-8) contains supplementary material, which is available to authorized users.

## Background

Telocytes (TCs) as a new type of interstitial cells are characterized with extensive telopodes [1], and found in multiple organs/tissues, including heart [2], trachea and lung [3], digestive tract [4, 5], liver [6], skeletal muscle [7], kidney and urinary tract [8, 9], skin [10], mammary gland [11], and others [12–18]. TCs were suggested to play a key role in supporting and nutrition of associated cells through the interconnection in forms of a complex three-dimensional network within organs/tissues [12, 19–21]. TCs are involved in mechanical support, intercellular signaling, immune surveillance, stem-cell guidance and tissue regeneration [22–24].

The electrophysiological properties of TCs were reported in recent studies [25]. Ion channels on the membrane of TCs are found to be related with the different function of TCs and are involved in multiple biological courses, e.g., SK3 channel modulates the contractility of myometrial [26], large conductance Ca^2+^-activated K^+^ current and inwardly rectifying K^+^ current are expressed on TCs in human heart [27], etc.

Our previous studies initially demonstrated that pulmonary TCs were allocated near the basement membrane of the bronchiolar epithelium, between airway smooth muscle cells, or in pulmonary interstitial space [3].Pulmonary TCs were inferred to play significant roles in lung diseases, e.g. involving in the course of repair after injury, contributing to the development of pulmonary infectious diseases and stimulating the proliferation of fibroblasts during the process of fibrosis, etc. [28]

TCs are mainly identified and defined by the structures of telopodes, podoms and podomers through transmission electron microscopy [4, 22]. CD34/PDGFRα double immunohistochemistry can orientate the diagnosis [29]. The identification course of TCs is relatively complicated and the confusion between TCs and other cells is inevitable. Therefore, there is a need to select several specific biomarkers of TCs to differ between pulmonary TCs and other tissue resident cells, e.g. fibroblasts, stem cells, epithelial cells, and inflammatory cells.

Our previous studies focused on TCs in lung and trachea of the mouse [3] provided genetic evidence that pulmonary TCs differ from stem cells and fibroblasts through comparing the variation of gene expression profiles [30]. Other studies suggested specific microRNA expression signatures as TC-specific biomarkers [31–33]. Our recent studies identified characters and patterns of TC-specific or TC-dominated gene profiles and fingerprints in chromosome 1, 2, 3, 17 and 18, through global comparison between TCs and other cells in the mouse lung tissue [34–36]. The present study aims to search TC-specific or dominated gene profiles and fingerprints of chromosome X, investigate the network of principle genes, and explore potential functional association. We globally compared gene expression profiles of TCs, mesenchymal stem cells (MSCs), fibroblasts (Fbs), alveolar type II cells (ATII), airway basal cells (ABCs), proximal airway cells (PACs), CD8^+^ T cells from bronchial lymphnodes (T-BL), and CD8^+^ T cells from lung (T-L), which may interact with TCs in the lung and trachea.

## Methods

### Isolation and primary culture of TCs from lung tissues

TCs were isolated from mouse lung tissues, cultured in a density of 1 × 10^5^ cells/cm^2^. TCs usually begin forming telopodes on day 5 and start overlapping and apoptosis on day 10, therefore TCs were harvested on day 5 (TC5) and day 10 (TC10), as described previously [30, 34]. RNAs were isolated and prepared, labeled, and hybridized for DNA microarray (The Mouse 4x44K Gene Expression Array, Agilent, Shanghai, China) with about 39,000+ mouse genes and transcripts represented with public domain annotations, according to the protocol of One-Color Microarray-Based Gene Expression Analysis. The hybridized arrays were washed, fixed and scanned with using the Agilent DNA Microarray Scanner (part number G2505B).

### Data collection and mining

We obtained gene expression profiles of pulmonary TCs on days 5 and 10, MSCs, Fbs from our previous study [30], ATII, ABCs, PACs, T-BL, and T-LL from the National Center for Biotechnology Information (NCBI) Gene Expression Omnibus database (GSE6846 [37], GSE27379 [38], GSE28651). The microarray was composed of 45,101 probes. Our first filter eliminated the probe sets without corresponding official symbol, leaving 39, 417 probes and 21,680 genes.

Our earlier study have made the gene expression profile, composed of 23,861 probes, of mouse lung TCs, Fbs and stromal stem/progenitor cells [30]. After eliminating the probes without corresponding official symbol, there are 13,236 probes and 11,532 genes. Only those genes whose expressions were measured in all cells were considered in our analysis. In total, 11,532 genes were analyzed and 335 genes of chromosome X were focused and furthermore analyzed in the present study.

### Identification of differentially expressed genes

The present study compared gene expression profiles in chromosome X of pulmonary TCs with MSCs, Fbs, ATIIs, ABCs, PACs, T-BL, or T-L by global analyses, investigated gene expression profiles of chromosome X in different cells to seek for the TC-specific regulated genes and explored potential association of selected genes.

**Fig. 1:**
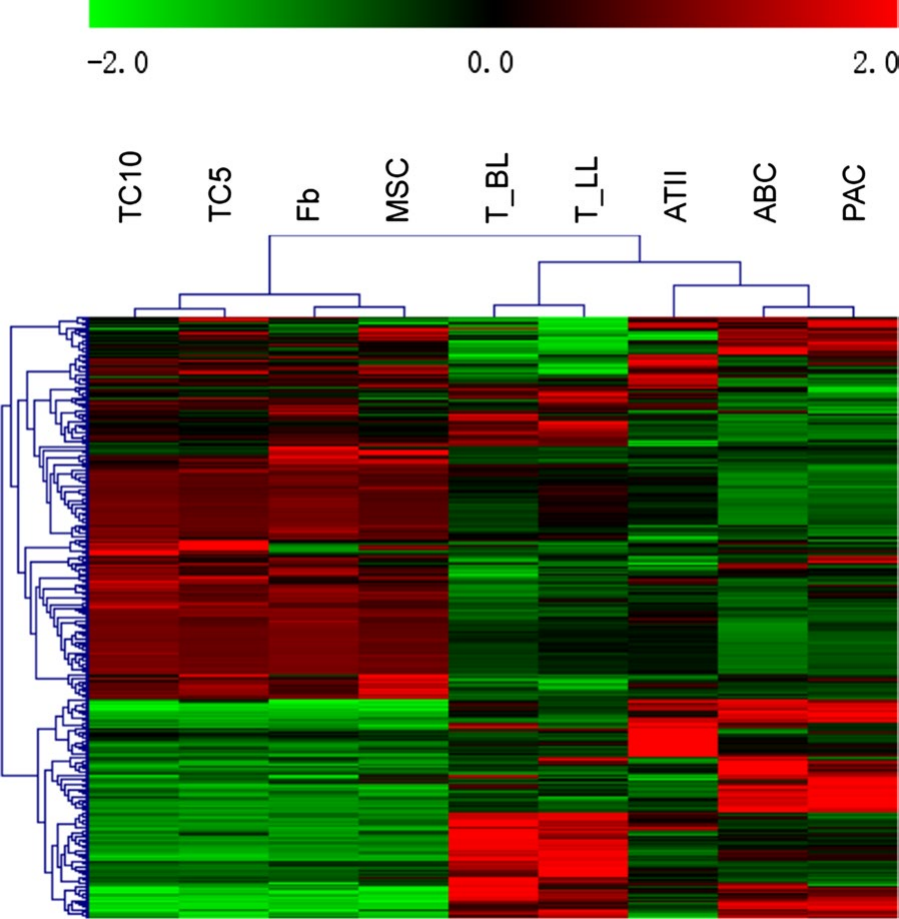
Hierarchical cluster analysis. Hierarchical cluster analysis of the differentially expressed genes on chromosomes X among telocytes (TCs), mesenchymal stem cells (MSCs), fibroblasts (Fbs), lymphocytes from lungs (T-LL) and from bronchial lymph nodes (T-BL), alveolar type II cells (ATII), proximal airway cells (PAC) and airway basal cells (ABC)

Hierarchical clustering of genes in chromosomes X was performed by TIGR Multi-experiment Viewer (MeV v4.9) (Fig. 1). Gene expression data were normalized and imported into Agilent GeneSpring GX software (version 11.5.1) for further analysis. Up- or down-regulated folds of TCs genes were calculated by comparing with other cells, after the averages of gene expression in cells were obtained from the raw data of multi-databases. The up-regulated folds were defined as the normalized gene expression values of TC5 or TC10 divide those of other cells, while the down-regulated folds were defined as the expression values of other cells devide those of TC5 or TC10. We selected the genes which were consistently up or down regulated (>1 fold) in both TC5 and TC10 as compared to other cells as TC-specific genes (Additional file 1: Table S1).

## Results

Hierarchical clustering of genes in chromosomes X was performed, as shown in Fig. 1. The result of clustering showed a close relationship of TC5 and TC10, and obvious difference between TCs and other kind of cells.

Gene expression array data showed that 8 chromosome X genes (e.g. Flna, Msn, Cfp, Col4a5, Mum1l1, Rnf128, Syn1, and Srpx2) up-regulated in both TC5 and TC10, as compared with those in other cells (Table 1). Of them, Cfp, Col4a5, Mum1l1, Rnf128, and Syn1 up-regulated 2–5 folds, and Srpx2 more than 5 folds as compared with other cells (Table 1B). 23 genes (e.g. Abcb7, Atf1, Ddx26b, Drp2, Fam122b, Gyk, Irak1, Lamp2, Mecp2, Ndufb11, Ogt, Pdha1, Pola1, Rab9, Rbmx2, Rhox9, Thoc2, Vbp1, Dkc1, Nkrf, Piga, Tmlhe and Tsr2) down-regulated in TCs, of which 5 chromosome X genes (Dkc1, Nkrf, Piga, Tmlhe, and Tsr2) in TCs were more than 2 folds lower than in other cells, as compared with other cells (Table 2).

**Table 1:** Summary of genes up-regulated in TCs, as compared with others

Compaired pairs/fold up-regulated	>1	>2	>5
TC5 vs others	24	9	1
TC10 vs others	12	8	2
TCs vs others	8	6	1

Gene symbol	Folds (TC5 vs others/TC10 vs others)						
	Fb	MSC	ATII	T_BL	T_LL	ABC	PAC
(A) Genes up-regulated between 1- and 2-folds in TCs as compared with others							
Flna	14.13/8.85	1.82/1.14	16.21/10.15	28.84/18.06	49.80/31.18	26.52/16.60	22.41/14.03
Msn	5.43/3.93	1.83/1.32	3.45/2.50	2.97/2.15	4.11/2.97	8.02/5.80	7.86/5.69
(B) Genes up-regulated between 2- and 5-folds in TCs as compared with other							
Cfp	6.05/8.22	7.53/10.23	2.15/2.92	113.12/153.60	9.46/12.85	41.52/56.38	36.31/49.30
Col4a5	11.55/9.00	10.47/8.15	5.20/4.05	313.12/243.83	81.54/63.50	2.89/2.25	7.92/6.16
Mum1l1	2.67/2.60	3.65/3.56	4.31/4.19	21.36/20.81	35.42/34.50	17.91/17.44	81.21/79.09
Rnf128	161.75/297.77	13.59/25.01	8.99/16.55	37.62/69.25	11.83/21.77	2.07/3.82	2.52/4.63
Syn1	34.19/35.13	7.52/7.72	6.66/6.84	9.60/8.86	3.26/3.35	3.36/3.46	2.77/2.85
(C) Genes up-regulated between 5- and 10-folds in TCs as compared with other							
Srpx2	32.62/54.91	20.66/34.78	82.00/138.03	100.82/169.72	55.79/93.92	6.25/10.52	23.63/39.77

**Table 2:** Summary of genes down-regulated in TCs, as compared with others

Compaired pairs/fold down-regulated	>1	>2	>5
TC5 vs others	41	6	0
TC10 vs others	91	22	1
TCs vs others	23	5	0

Gene symbol	Folds (TC5 vs others/TC10 vs others)						
	Fb	MSC	ATII	T_BL	T_LL	ABC	PAC
(A) Genes down-regulated between 1- and 2-folds in TCs as compared with others							
Abcb7	1.37/1.97	1.50/2.15	17.81/25.48	26.27/37.59	15.70/22.47	26.13/37.39	23.93/34.24
Atf1	1.89/2.00	1.86/1.97	6.57/6.95	11.93/12.62	13.90/14.70	23.09/24.43	17.32/18.32
Ddx26b	1.32/1.22	4.39/4.06	5.57/5.15	8.24/7.62	14.11/13.05	4.48/4.14	2.93/2.71
Drp2	1.26/1.79	1.43/2.04	2.58/3.67	2.32/3.30	4.42/6.29	3.67/5.22	2.11/3.01
Fam122b	5.05/6.22	1.08/1.32	1.52/1.87	4.03/4.96	2.87/3.53	6.53/8.03	1.59/1.96
Gyk	2.01/1.79	1.59/1.42	24.83/22.14	6.30/5.62	8.25/7.36	5.35/4.77	16.66/14.85
Irak1	1.31/1.28	1.70/1.66	4.59/4.50	6.03/5.90	5.61/5.49	5.66/5.54	3.52/3.45
Lamp2	1.23/1.36	2.07/2.30	3.91/4.34	1.02/1.14	1.31/1.45	4.49/4.99	3.49/3.88
Mecp2	1.59/1.84	2.35/2.72	1.98/2.29	3.26/3.77	2.79/3.23	2.60/3.01	1.48/1.71
Ndufb11	1.34/1.80	1.05/1.42	4.64/6.26	3.74/5.05	2.71/3.65	5.41/7.30	4.84/6.53
Ogt	1.48/1.63	5.10/5.64	3.73/4.11	15.00/16.56	14.45/15.96	10.01/11.06	6.00/6.63
Pdha1	1.08/1.13	1.16/1.21	9.30/9.73	14.59/15.28	12.53/13.11	10.88/11.39	10.18/10.66
Pola1	1.45/2.04	1.30/1.83	1.31/1.84	1.96/2.76	1.48/2.09	1.69/2.38	2.71/3.82
Rab9	1.03/1.14	1.53/1.70	5.01/5.56	2.89/3.21	2.83/3.14	2.65/2.95	2.84/3.15
Rbmx2	1.01/1.54	1.85/2.81	4.23/6.43	14.71/22.35	10.64/16.17	8.91/13.54	9.89/15.03
Rhox9	1.49/1.53	1.12/1.15	13.98/14.39	13.94/14.35	20.54/21.15	21.07/21.69	8.39/8.64
Thoc2	1.40/1.66	2.54/3.00	1.83/2.17	10.15/12.00	12.25/14.49	7.73/9.14	6.27/742
Vbp1	1.04/1.22	1.44/1.70	4.70/5.53	17.42/20.48	16.14/18.98	7.87/9.26	7.10/8.35
(B) Genes down-regulated between 2- and 5-folds in TCs as compared with others							
Dkc1	2.05/2.62	3.58/4.57	8.45/10.78	20.66/26.35	14.18/18.09	28.20/35.98	38.11/48.61
Nkrf	2.36/2.47	2.38/2.50	10.62/11.12	25.28/26.46	14.63/15.32	10.10/10.57	10.10/10.57
Piga	3.52/4..74	3.16/4.25	2.05/2.75	3.41/4.59	3.83/5.16	3.24/4.36	13.95/18.76
Tmlhe	2.62/5.24	3.80/7.61	2.47/4.94	11.75/23.51	8.01/16.03	10.56/21.13	9.27/18.55
Tsr2	3.65/3.27	3.22/2.89	24.69/22.09	24.70/22.10	23.11/20.69	33.31/29.81	26.16/23.41

A set of genes were found specifically up- or down-regulated in pulmonary TCs, as compared with MSCs, Fbs, ATII, ABCs, PACs, T-BL, or T-L, respectively, as listed in Table 3. A set of genes up- or down-regulated more than one fold in TC5 were 178 or 157, 224 or 111, 101 or 234, 99 or 236, 109 or 226, 68 or 267, or 88 or 247 and in TC10 123 or 212, 180 or 155, 75 or 260, 87 or 248, 86 or 249, 51 or 284, or 71 or 264, as compared with MSCs, Fbs, ATII, T-BL, T-L, ABCs, PACs, respectively. Up- or down-regulated genes in both TC5 and TC10 were 119 or 153, 172 or 103, 72 or 231, 79 or 228, 81 or 221, 43 or 259, or 65 or 241, as compared with MSCs, Fbs, ATII, T-BL, T-L, ABCs, PACs, respectively. Details of up- or down- regulated gene variations of chromosome X were listed in Additional file 2: Table S2, including the number and names of up- or down-regulated genes among different cells.

**Table 3:** The number of genes specifically up- or down-regulated in pulmonary telocytes, as compared with other cells

Compared pairs	Up >1	Up >2	Up >5	Down >1	Down >2	Down >5	Down >10
TC5 vs MSC	178	58	21	157	56	9	3
TC10 vs MSC	123	49	18	212	92	14	8
TCs vs MSC	119	36	13	153	45	9	3
TC5 vs Fb	224	100	65	111	28	9	6
TC10 vs Fb	180	81	34	155	65	14	7
TCs vs Fb	172	68	29	103	25	8	5
TC5 vs ATII	101	47	19	234	167	85	42
TC10 vs ATII	75	38	15	260	199	102	56
TCs vs ATII	72	33	12	231	163	73	40
TC5 vs T_BL	99	68	46	236	168	90	54
TC10 vs T_BL	87	59	36	248	201	109	66
TCs vs T_BL	79	54	34	228	166	85	52
TC5 vs T_LL	109	66	35	226	176	103	56
TC10 vs T_LL	86	56	31	249	201	122	75
TCs vs T_LL	81	54	27	221	172	98	55
TC5 vs ABC	68	40	14	267	210	121	67
TC10 vs ABC	51	31	14	284	230	146	90
TCs vs ABC	43	28	11	259	199	116	65
TC5 vs PAC	88	47	22	247	180	89	46
TC10 vs PAC	71	43	20	264	210	111	67
TCs vs PAC	65	37	16	241	174	84	44

**Fig. 2:**
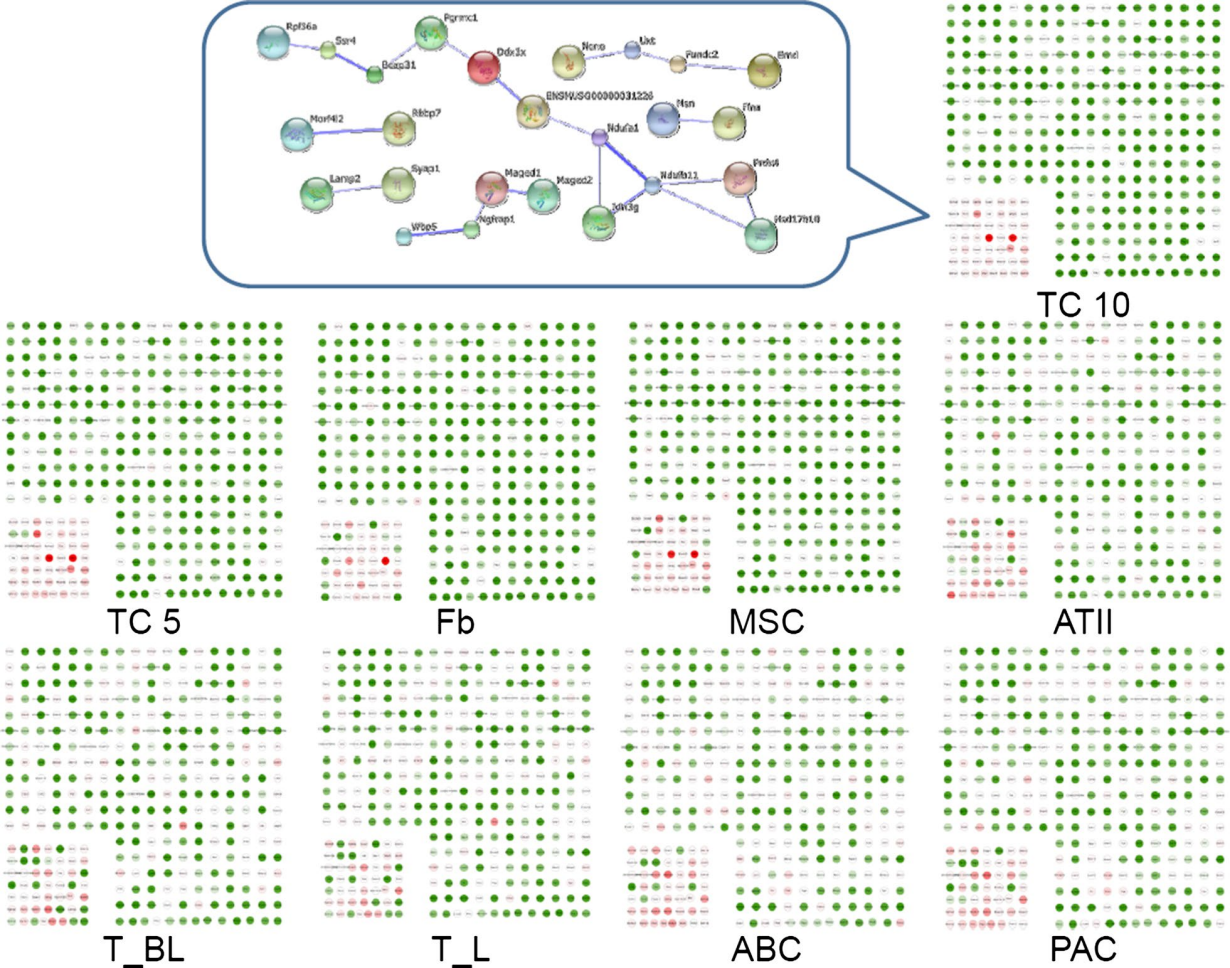
Expression profiles of the top 15 % up-regulated genes of chomosome X in TCs. Expression profiles of the top 15 % up-regulated genes of chromosome X of TCs isolated and cultured from mouse lungs on days 10 (TC10), as compared with those on days 5 (TC5), MSCs, Fbs, ATII, ABCs, PACs, T-BL, and T-L. The profiles for entire genes are described in Additional file 1. The selected core network and whole mouse network are linked by the documented functional inte*red*ractions from various databases (see “Methods”). Genes in each network are indicated in and some of their nearest neighbors are indicated by *grey* nodes. The top 15 % up-regulated genes within chromosome X of TC10 were selected, and their distribution in each type of cells was compared, and showed the difference between cells

We picked out the top 15 % high-expressed genes in chromosome X of TC10, and compared the distribution of such active gene group with other cells, as shown in Fig. 2. The distribution of the high expressed genes in TC10 was similar to those in TC5, while quite different from MSCs, Fbs, ATII, ABCs, PACs, T-BL or T-L. These high expressed genes are mostly involved in gene transcription, energy metabolism, apoptosis and protein degradation, cell migration and intracellular trafficking, and DNA repair. The relationships, including direct (physical) and indirect (functional) associations, of these genes were analyzed by String Network analysis (http://www.string-db.org). The interaction and potential functional links of those genes are also displayed in Fig. 2.

**Fig. 3:**
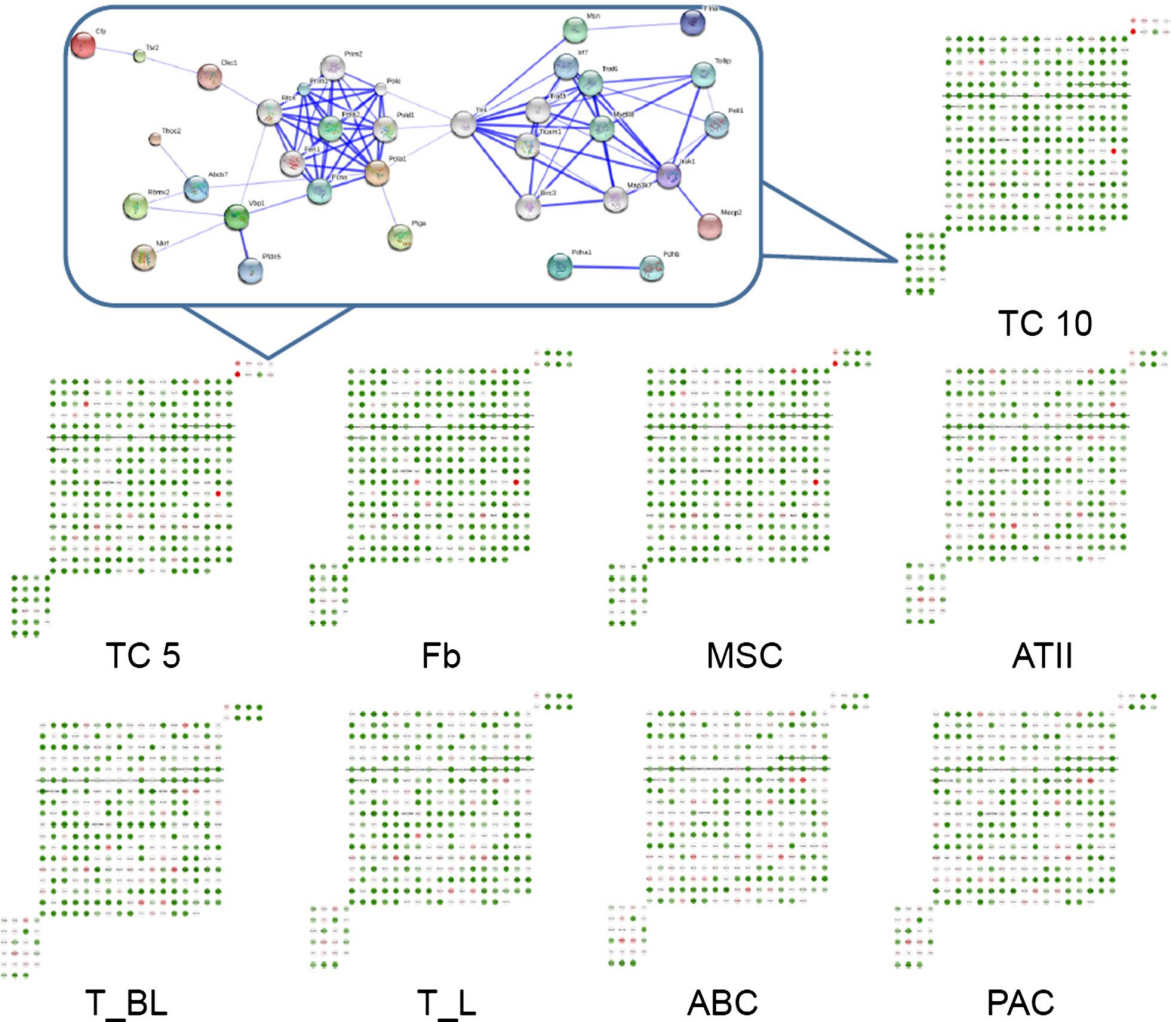
Expression profiles of TC-specific or dominant genes. Expression profiles of the up- or down-regulated genes (more than one fold) in both TC5 and TC10 as compared with MSCs, Fbs, ATII, ABCs, PACs, T-BL and T-L. The profiles for entire genes are described in Additional file 1. The selected core network and whole mouse network are linked by the documented functional interactions from various databases (see “Methods”). Genes in each network are indicated in *red* and some of their nearest neighbors are indicated by *grey* nodes. The 8 up-regulated genes in TCs were shown in the *upper right corner*, while the 23 down-regulated genes in the *lower left* and their distribution in each type of cells showed in Fig. 2b. These up- or down-regulated genes were considered as TC-specific or TC-dominant genes. The connections between these genes were analyzed and illustrated

Thirty-one genes were selected as TC-specific or dominated genes, which were up- or down-regulated more than one fold in both TC5 and TC10, as compared with other cells. Among those genes in TCs, 8 were up-regulated, while 23 were down-regulated. Figure 3 demonstrated the distribution of such distinct TC-specific or dominated genes in chromosome X of all cells. The interaction and potential functional links between those genes are also displayed in Fig. 3.

## Discussion

**Fig. 4:**
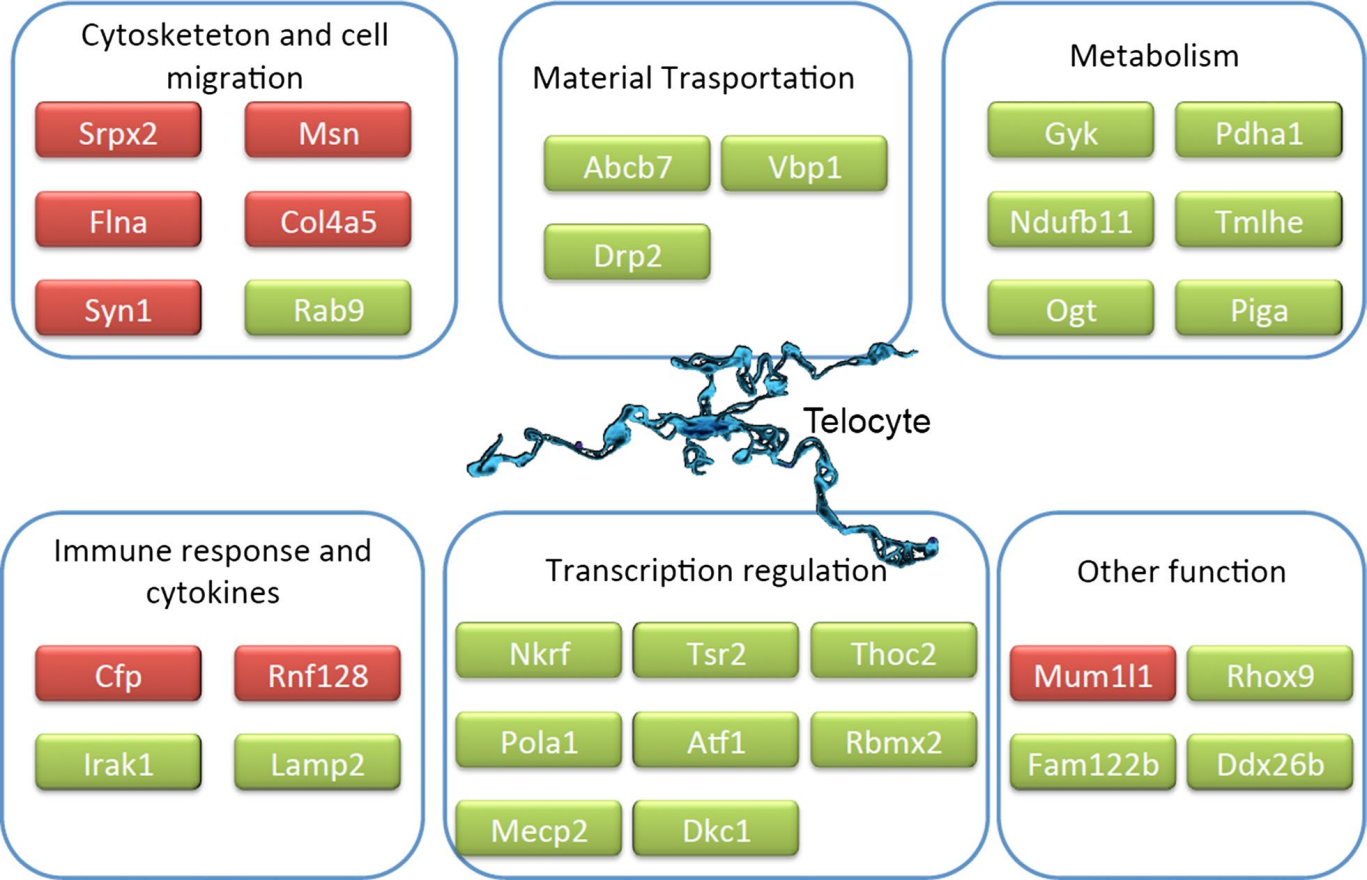
TC-specific or dominant genes and their main function. Gene expression array data showed that 8 chromosome X genes up-regulated in both TC5 and TC10, and 23 genes down-regulated, as compared with those in other cells. These genes are selected as TC-dominant or specific genes. The up-regulated genes showed in *red color* while the down-regulated genes in *green color*. The up-regulated genes are mostly involved in cytoskeleton and cell migration, and regulation of immune responses. The down-regulated genes participates mainly in gene transcription modulation, energy metastasis, material transportation and so on

Chromosome X is one of the two sex-determining chromosomes, which exists in both gender, with one copy in male and two copies in female, spanning about 156 million base pairs and 1805 genes in human cells. Although chromosome X contains only 4 % of all human genes, a large number of disease condition are related with chromosome X, including X-linked diseases and 10 % of diseases with a mendelian pattern of inheritance [39]. There are 20,000–25,000 genes in mouse, and the similarity of genes between human and mouse is about 85 %. Among 2059 genes in chromosome X of the mouse, 335 were measured by bioinformatics tools in the present study. There were about 8 or 23 up- or down-regulated genes of chromosome X in TCs, as compared with stem cells, lung interstitial cells, pneumocytes, airway cells, or lymphocytes (Fig. 4).

Srpx2, Cfp, Col4a5, Mum1l1, Rnf128 and Syn1 over-expressed in chromosome X of TCs, as compared with other cells. Srpx2 (sushi-repeat-containing protein, X-linked 2) gene was the only one of chromosome X that expressed more than 5 folds in pulmonary TCs as compared with all the other cells. The Srpx2-encoded protein was found to be the key factor in the development of speech and language centers in the brain [40], the important mediator in endothelial cell migration and tube formation, and the predictor for tumor growth and metastasis [41–43]. The enrichment of Srpx2 in pulmonary TCs suggests the potential role of TCs in the regulation of neuron function, promotion of vessel formation, and enhance of cellular growth and migration. Those could be performed through the complicated three-dimensional network constructed by the telopodes with other cells. Cfp, Col4a5, Mum1L1, Rnf128 and Syn1 over-expressed 2–5 folds in TCs within the lung tissue. Cfp (complement factor properdin) encodes a plasma glycoprotein that positively regulates the alternative complement pathway of the innate immune system [44]. TCs were found to be associated with the immune cells including lymphocytes [45], basophiles [46], eosinophils and plasma cells [7]. The high-expressed Cfp suggested that TCs might participate in immune responses and play a potential role in the lung infectious diseases. Col4a5 (collagen, type IV, alpha 5) encodes type IV collagen, which is the major structural component of basement membranes and is critical for tissue remodeling and repair [47]. The Col4a5 gene was also found as a TC-dominant gene as compared with Fbs or MSCs in our previous study [30].

Rnf128 (ring finger protein 128, E3 ubiquitin protein ligase) is associated with cellular apoptosis and cytokine regulation, of which over-expression inhibits the production of activation-induced IL2 and IL4 cytokine, and may converse T cells to the anergic phenotype [48, 49]. This implies that TCs may be involved in inflammation, immune surveillance, or tissue repair after injury by regulating immune function and inflammatory cell. Syn1 (synapsin I) encodes neuronal phosphoproteins associated with the cytoplasmic surface of synaptic vesicles, by which TCs may establish synapses as a special type of heterocellular junctions with other neighbouring cells, including lymphocytes, macrophages, or mast cells [46, 50, 51]. Through those connections to immune cells, TCs could integrate signals, and behave as an immune system modulator. Mum1l1 (melanoma associated antigen (mutated) 1-like 1) is a gene related with melanoma or some other tumors, its function in TCs is not quite clear yet.

Genes down-regulated in chromosome X of pulmonary TCs mainly contribute to gene transcription, protein synthesis and energy metabolism. Nkrf (NFKB repressing factor) encodes a transcriptional repressor of nuclear factor kappa B (NF-κB) and is activated for cell survival, proliferation and immune responses. It indicates that TCs per se have relatively high capacities of proliferation and involvements of immune reactions in physiological conditions, although there are needs of direct evidences to show roles and changes of TCs in cancers, infection, autoimmune diseases, or injury [52–55]. Tsr2 [TSR2, 20S rRNA accumulation, homolog (*S. cerevisiae*)] is another inhibitor of NF-κB, and induces cell apoptosis [56]. Pulmonary TCs may be more involved in activities and processes dominanted by NF-κB such as the production of inflammatory mediators, due to the down-regulation of NF-κB inhibitors in TCs. Dkc1 (dyskeratosis congenita 1, dyskerin) plays an important role in telomerase stabilization and DNA damage response, regulation of a subset of microRNAs, nucleo-cytoplasmic shuttling, and cell adhesion [57]. It suggested that TCs have better abilities to resist senescence and external stresses. Piga (phosphatidylinositol glycan anchor biosynthesis, class A) is a gene related with paroxysmal nocturnal hemoglobinuria and encodes a protein required for synthesis of *N*-acetylglucosaminyl phosphatidylinositol, the first intermediate in the biosynthetic pathway of a glycolipid on blood cells and to anchor proteins to the cell surface [58]. Tmlhe (trimethyllysine hydroxylase, epsilon) encodes the first enzyme in the carnitine biosynthesis pathway related with the transport of activated fatty acids across the inner mitochondrial membrane [59]. The significance of those two down-regulated genes in TCs still remains unclear. Based on the level of gene expression, the limitation of the study is inevitable, since gene expression level cannot equally reflect the protein level or the function change in the cells. Fold changes of gene expression may not be in direct proportion to the functional significance. More studies will be conducted in the future to detect the relationships among TC-specific genes, TC-specific proteins and the function of TCs.

## Conclusions

In conclusion, the present study initially compared genetic variations of chromosome X of pulmonary TCs with MSCs, Fbs, ATIIs, ABCs, PACs, or bronchial and lung lymphocytes by global analyses. Our data demonstrated that 8 or 23 genes of TC chromosome X up- or down-regulated, respectively, indicating that biological functions of TCs may be mainly fulfilled through the network constructed by the telopodes with connecting cells and act as an integrated modulator to regulate neural function and immune responses, promote vessel formation and cellular growth and migration, and participate in tissue remodeling and repair. TCs may have the potential function to support cell survival, anti-senescence, and protection from stresses. Pulmonary TCs with stronger NF-kB-dominant activities may play the important and critical roles in pathogeneses of lung diseases, although further studies on those chromosome X genes of pulmonary TC are needed.

## Additional files

**Additional file 1.** Data profiles for all genes in chromosome X.
**Additional file 2.** Details of up-regulated or down-regulated gene expression variations in chromosome X.

## Abbreviations

TC: telocyte
TC10: telocytes isolated and cultured from mouse lungs on days 10
TC5: telocytes isolated and cultured from mouse lungs on days 5
MSC: mesenchymal stem cell
Fb: fibroblast
ATII: alveolar type II cell
ABC: airway basal cell
PAC: proximal airway cell
T-BL: CD8^+^ T cells come from bronchial lymph node
T-L: CD8^+^ T cells from lungs
